# The Hygiene Hypothesis and New Perspectives—Current Challenges Meeting an Old Postulate

**DOI:** 10.3389/fimmu.2021.637087

**Published:** 2021-03-18

**Authors:** Holger Garn, Daniel Piotr Potaczek, Petra Ina Pfefferle

**Affiliations:** ^1^ Translational Inflammation Research Division & Core Facility for Single Cell Multiomics, Medical Faculty, Biochemical Pharmacological Center (BPC), Philipps University of Marburg, Marburg, Germany; ^2^ German Center for Lung Research (DZL), Marburg, Germany; ^3^ Comprehensive Biobank Marburg (CBBMR), Medical Faculty, Philipps University of Marburg, Marburg, Germany; ^4^ German Biobank Alliance (GBA), Marburg, Germany

**Keywords:** hygiene hypothesis, allergy, asthma, non-communicable inflammatory diseases, chronic inflammation

## Abstract

During its 30 years history, the Hygiene Hypothesis has shown itself to be adaptable whenever it has been challenged by new scientific developments and this is a still a continuously ongoing process. In this regard, the mini review aims to discuss some selected new developments in relation to their impact on further fine-tuning and expansion of the Hygiene Hypothesis. This will include the role of recently discovered classes of innate and adaptive immune cells that challenges the old Th1/Th2 paradigm, the applicability of the Hygiene Hypothesis to newly identified allergy/asthma phenotypes with diverse underlying pathomechanistic endotypes, and the increasing knowledge derived from epigenetic studies that leads to better understanding of mechanisms involved in the translation of environmental impacts on biological systems. Further, we discuss in brief the expansion of the Hygiene Hypothesis to other disease areas like psychiatric disorders and cancer and conclude that the continuously developing Hygiene Hypothesis may provide a more generalized explanation for health burden in highly industrialized countries also relation to global changes.

## Introduction

Throughout its history, the Hygiene Hypothesis has shown itself to be adaptable and flexible whenever it has been challenged by innovation in science (1). A number of new findings need to be considered in this ongoing revisiting process: The originally proposed Th1/Th2 paradigm is challenged by currently elucidated new classes of effector and regulating immune cells pointing out to a more complex immune network involved in allergy development (2). Studies on biomarkers and deep phenotyping techniques changed our understanding of asthma as a uniform disease in favor of distinct phenotypes that are driven by different causations (3). The emerging field of epigenetics enables us to fill the black box of “gene-by-environment interactions” with conveying mechanisms (4). Currently recognized epigenetic pathways overlapping between chronic inflammatory diseases and other disorders such as psychiatric conditions or cancer might extend the Hygiene Hypothesis toward a model explaining in a broader sense the rise of health burdens in westernized societies (5). Finally, the world-wide challenge caused by the climate changes will not leave the consequences of Hygiene Hypothesis unaffected. Changing life-styles are closely related to measures implemented to slow down CO_2_ emissions and to stabilize the world climate (6).

## Challenges From Immunology – The Immune System Becomes More Complex

Parallel to the revisions that Hygiene Hypothesis has undergone over time (7), our perception of the mechanisms underlying cellular and humoral immune responses has changed fundamentally over the last decades. High-resolution flow cytometry and cell sorting and, most recently, single cell multiomics-based analyses provided a deeper insight into the phenotypic characterization, function, and development of diverse classes of hematopoietic cell types. The dichotomous model of divergent Th1 and Th2 responses was significantly expanded by the discovery that T lymphocytes represent a branched network of subsets, characterized by a high level of plasticity and adaptability (8). Namely, Sakaguchi’s discovery of regulatory T-cell (Treg) subsets provided a significant new impetus to researchers investigating the immunological origin of allergic and autoimmune diseases and their prevention under healthy conditions and pointed out new strategies to combat those maladies (9, 10). Moreover, the discovery of new classes of effector cells and their cellular interactions added relevant evidence to the field. As one example, innate lymphoid cells (ILC) became of main interest as they have been shown to be both directly and indirectly associated with and involved in the development of allergic responses (11). This unique class of effector cells lacks a clonally distributed antigen receptor which thus resemble innate immune cells characterized by (antigen) unspecific activation, however, they exert T helper (Th)-like effector cell activities (12). According to their expression of effector cytokines and transcription factors ILC have been classified into three groups: ILC1, ILC2, and ILC3 (13). While ILC1 produce interferon-gamma (IFNγ) and tumor necrosis factor α (TNFα) and, similarly to Th1 cells, express T-bet, ILC2 are able to produce Th2 cytokines such as IL-5 and IL-13, like Th2 cells under the control of the transcription factor GATA-3. ILC3 are similar to Th17 cells and release IL-17A and IL-22 as well as granulocyte macrophage colony stimulating factor (GM-CSF). In animal models of allergic airway inflammation as well as in human allergic asthmatics ILC2 are present at elevated frequencies within the lung and airways epithelial compartments where they were found to produce high amounts of the type-2 cytokines IL-5 and IL-13 (14). Within the last years, ILC2 have been recognized as early promoters to establish and maintain allergic airway inflammatory responses but also as protectors promoting repair processes of the lung epithelium (15, 16).

A potential link between the Hygiene Hypothesis and the function of ILC lineages comes from the gut. The symbiotic interaction between immune cells and the microbiota in the gut is principally decisive for the development of tolerance or pathogenicity. The ILC3 lineage is essential in the development of lymphoid follicles and Peyer’s patches in the gut and was shown to be crucial for the maintenance of a well-balanced symbiosis with the microbiota (17). The host microbiota itself might play an important role in determining ILC subsets specificity as indicated by results coming from experimental approaches. Sepahi et al. very recently reported that short chain fatty acids (SCFA) arising from dietary fibers by microbial fermentation in the intestine induced expansion of prevailing ILC subsets. By triggering ILC subset expansion *via* G-protein-coupled receptors (GPCR) those dietary metabolites contribute to the homeostasis in the local compartment (18). Another mechanism to induce repair and homeostatic conditions at epithelial surfaces is mediated *via* IL-22-producing ILC3 in response to the microbiota. In interaction with IL-18 produced by the epithelial cells, IL-22 is involved in the promotion of repair and remodeling processes as well as in the maintenance of the gut homeostasis (19). By acting as mediators between the microbiota and the host ILC are recognized as crucial in the early host response to microbial stimuli.

## Challenges From Changing Environments – Can Epigenetics Provide the Missing Link to Explain “Gene-by-Environment Interactions”?

Very recently, damaging factors that jeopardize the normal development or disturb the balance of an established immune system have come into the focus of research on allergic diseases. Environmental changes caused by in- and outdoor pollution (20, 21) and the global warming impact the atopic epidemic and some attempts were undertaken recently to integrate these scenarios into the concept of the Hygiene Hypothesis on the basis of epigenetic changes driven by gene-by-environment interactions (22).

In contrast to our ancestors who spent most of their life time outdoors and thus close to a natural environment, post-modern and mainly urban life-styles are characterized by a significantly higher proportion of indoor activities. These changing habits underline the potential importance of indoor air composition on the development of allergic diseases and further emphasize the role of the environmental microbiota (23). Indoor air in urban homes is often burdened with elevated levels of molds which are found to be harmful to the airways and favor the development of airway inflammation and asthma (24). Against the background of growing climate awareness and the resulting increased efforts to reduce energy consumption and CO_2_ emissions, current research on these indoor exposures in homes with improved house insulation points out to an up-coming health problem. Enrichment of volatile organic compounds released from furniture or brought in by tobacco smoke as well perennial allergens and molds will jeopardize mainly infants as the developing immune system and the growing lung are highly susceptible to these damage factors (25). Already the fetus might become affected by these components (26). This was exemplarily shown for tobacco smoke in a transgenerational case control study conducted to assess the risk for asthma by prenatal smoking. Grandmothers and mothers of asthmatic and non-asthmatic children were asked about smoking habits during their own pregnancy. The study reported an odds ratio twice as high for children to develop asthma in families where grandmothers frequently smoked during the mother’s fetal period (27).

At that point the Hygiene Hypothesis was in line with an upcoming general idea that non-inherited/non communicable diseases like allergies and asthma develop on the background of an inappropriate interaction between environmental exposures and a given genotype to shape a specific (disease) phenotype. Though based on the concept of a so-called epigenetic landscape postulated by Waddington already in the 50ties of the last century, the underlying molecular mechanisms of epigenetic programming had still been the “missing link” in the scenario of gene-by-environment-interactions (28). By discovering mechanisms such as DNA methylation, diverse histone modifications and microRNA regulation as molecular mechanisms underlying epigenetic regulation of gene expression, an exciting new field of research was opened that currently has a strong impact on research aiming to unravel the still existing mysteries of allergy development and prevention (29–31).

Indeed, epigenetic mechanisms have meanwhile clearly been demonstrated to be involved in mediating the effects of environmental factors increasing or decreasing the risk of allergy development (4). Pro-allergic environmental influences can be exemplified by pollution. For instance, higher *in utero* exposure to polycyclic aromatic hydrocarbons (PAH) has been shown to be associated with increased cord blood leukocyte DNA methylation at the promoter of the IFNγ-encoding gene (32, 33). Moreover, in Treg isolated from peripheral blood mononuclear cells, higher PAH exposure has been correlated with elevated DNA methylation at the promoter of the gene encoding FOXP3, a master regulator of Treg development and activities, with the effect being stronger in asthmatic than in non-asthmatic children (34).

After epidemiological studies had demonstrated an association between spending early life time in specific agricultural environments and protection against the development of allergies in childhood (35, 36), functional investigations of various types started to clarify which elements of farming, such as contact with farm animals, consumption of raw cow’s milk, exposure to so-called farm-dust, and others, mechanistically underlie this observation. DNA demethylation at the FOXP3-encoding locus related to higher expression of the gene and activation of Treg (37) has been associated in cord blood with maternal consumption of raw cow’s milk (38) and in children’s whole blood with early-life ingestion of raw cow’s milk (39). Compared to processed shop milk, pretreatment with raw cow’s milk reduced features of the disease in mice subjected to a model of food allergy and this effect was mediated by changes in histone acetylation patterns at crucial T cell-related genes (40, 41). Interestingly enough, unprocessed cow’s milk has been shown to contain miRNAs potentially affecting the expression of important allergy-related immune genes, which might contribute to its protective effects against asthma (42). Several bacteria have been isolated from the farming environment, for instance *Acinetobacter lwoffii* (*A. lwoffii*), which were demonstrated to diminish the development of allergic symptoms in murine models (43). *A. lwoffii*-mediated protection against allergic airway inflammation has been observed in mouse models also transmaternally and shown to be IFNγ−dependent, with this effect being at least partly mediated by preservation of histone H4 acetylation at the promoter of the IFNγ-encoding gene as observed in CD4^+^ T cells isolated from spleens of the offspring (44, 45).

## Challenges From the Clinics and Lessons From Animal Models – Asthma Phenotypes and the Hygiene Hypothesis

A recurrent debate flared up in the field of asthma research excellently summarized at the time being in a review by Wenzel in 2012 (46). Coming from clinical heterogeneity of asthma patients she highlighted that basic inflammation patterns differ in asthma patients which in turn determines the success of the applied therapeutic strategy. As an early diagnosis and adequate treatment may prevent the development of a severe asthma phenotype later on, novel strategies to discriminate children at risk from those who will not develop asthma are required (47). Following the clinical definition of a phenotype as a result of an interaction between a given genotype and the environment Wenzel and colleagues expressed the strong medical need for novel molecular and genetic biomarkers indicative for the characterization of such phenotypes and defining the specific requirements for stratified therapies. Based on differences between Th2-driven atopic asthma and non-atopic asthma a number of subtypes were defined that evolve and differ with age and respond differentially to standard drug treatment regimes. It quickly became clear that the search for a specific biomarker that clearly identifies a respective phenotype would not be successful. Rather, the synopsis of all data collected from a subject known as “deep phenotyping” may lead to better understanding of complex asthmatic conditions (48). Deep phenotyping in the era of OMICS goes along with a tremendous increase in data that needs to be analyzed. To handle these big data-sets new approaches become increasingly employed involving models of statistical data dimension reduction and machine-learning strategies (49, 50). The idea behind these data-driven approaches is to mine data collections and classify them based on so far hidden patterns behind the data. The hypothesis-free latent class analysis (LCA) approach represent one of the most promising tools to identify new or verify proposed asthma (and other allergic disease) phenotypes. A first LCA approach was carried out in two cohorts of adult asthmatics. Based on clinical and personal characteristics Siroux et al. described two distinct phenotypes in two independent cohorts, a severe phenotype in which asthma is already established in childhood and a second type that starts in adulthood with milder outcomes (51). In line with the Hygiene Hypothesis, these results pointed out specific preconditions in infant age which pave the pathway to severe asthma later in life. LCA analyses in children substantiated the link between early onset and later disease since early clinical signs such as current unremitting wheezing episodes are ascribed to indicate a higher risk for asthma development later in life while transient wheezing seems to have no pathological consequences (52).

LCA approaches using data from patient studies elucidated that there might be phenotypic asthmatic manifestations that could be explained by the Hygiene Hypothesis while other phenotypes that might have different pathomechanistic origins failed to be covered by this supposition (53). Among others, this discrepancy led to new approaches in pre-clinical animal-based experimental set-ups as well as investigations based on human data. New animal models were employed to prove the postulate of such phenotypes that can be discriminated on the immunological and histological levels. By switching from the well-established Ovalbumin (OVA) model, where the sensitization was mainly achieved by a rather artificial intraperitoneal allergen sensitization in the presence of the type-2 driving adjuvant alum, to a more flexible administration of standardized house dust mite extracts (HDM) *via* the nasal route, it was feasible to induce a more natural and broader spectrum of inflammatory phenotypes ranging from typical allergic eosinophil-dominated respiratory inflammation to airway inflammatory conditions almost exclusively dominated by the influx of neutrophils (54–56). Such more flexible model systems allow deeper and more precise investigations of the mechanisms underlying the development of different phenotypes and a much better characterization of the orchestration of different regulatory and effector T cell subsets in dependence of allergen administration on a continuum between Th2 and Th1/Th17-driven inflammation. In addition, these mouse models mimic the natural situation more closely by using common allergens and a potential natural route of sensitization and thus became helpful for understanding the diverse clinical phenotypes of allergic and non-allergic as well as mild and severe asthma (57, 58). By switching between different effector T cell responses in these experimental set-ups substantial knowledge is currently added to our understanding of clinical manifestations in asthma. In combination with LCA helping to elucidate clinical phenotypes these recent research developments strongly boosted a better discrimination between transient and persistent pediatric allergic conditions as well as allergic and non-allergic asthma later in life. This new evidence might lead us to the current limits of the Hygiene Hypothesis. While IgE-driven allergic asthma undoubtedly fits to the Hygiene Hypothesis, it is still unclear whether this holds true also for non-atopic asthma phenotypes the development of which is much more strongly determined by factors different from a missing (microbial) education of the immune system. Thus and to further fine-tune the Hygiene Hypothesis, continuous efforts are required to distinguish between environmental conditions (such as early life infection with pathogenic viruses) that are either associated with the induction of a disease phenotype and/or just contribute to a shift between distinct inflammatory manifestations of allergic disease phenotypes (59, 60) and those that really result in a general or a phenotype/endotype-specific prevention of disease in line with the Hygiene Hypothesis (43, 61).

## Challenges From a View Over the Fence – The Hygiene Hypothesis in Psychiatric Disorders and Cancer

The French scientist Bach was the first who made the principal observation of a general inverse correlation in the prevalences of infectious versus non-communicable chronic inflammatory diseases within the last seven decades (62). Meanwhile we know that abundant exposure to a high diversity of infectious or even harmless microbes resulting in repeated, low-grade acute inflammatory episodes in early life, associates with lower prevalence of chronic inflammatory disorders accompanied by low levels of inflammatory markers in adulthood. Conversely, high levels of hygiene during perinatal and early childhood developmental periods characteristic for Western countries corresponds to higher levels of inflammatory markers correlating with a higher prevalence of chronic inflammatory disorders later in life. Based on these facts, it has been hypothesized that frequent episodes of low-grade, in most cases clinically symptom-free inflammation in infancy may balance responses to inflammatory stimuli and thus reduce the rate of continuation of chronic inflammation into adulthood, most probably by adequately shaping the adaptive immunity-dependent regulation (23).

Interestingly, this observation considers a broader spectrum of chronic inflammatory conditions beyond allergies that might fit under the umbrella of the Hygiene Hypothesis such as multiple sclerosis, irritable bowel disease or diabetes type 1 (63). Moreover, within the last years a similar approach emerged to explain the tremendous increase in psychiatric disorders in westernized countries. Mainly affective disorders such as major depression and bipolar disorder are increasingly diagnosed in the westernized world. Patients suffering from affective and anxiety disorders depict an array of features that mirror inflammatory conditions such as pro-inflammatory cytokines in the blood and the central nervous system accompanied by elevated levels of circulating C-reactive protein (CRP), activation of lymphocytes and inflammatory cellular signaling pathways (MAPK and NF-κB), with the question of causality remaining a chicken or egg problem (64). Nevertheless, based on genetic predispositions and epigenetic modifications in the brain (nervous system) and the periphery (immune system), both kinds of pathologies, mood and inflammatory disorders, might become established on the basis of a disturbed homeostasis of otherwise tightly balanced adaptive systems of the body. Interestingly but fitting to the hypothesis, the microbiota of the gut seems to play a critical role also in the development of psychiatric disorders as shown by recently conducted studies (65). Based on an interplay between the gut and the central nervous system, persistent stress and maltreatment modifies the nervous system and thereby the endocrine hypothalamic pituitary axis (HPA) which in turn alters gut microbiota by cortisol release (66). Dysbiosis in the gut might lead to a compromised cytokine balance in the blood followed by an activation of the microglia in the brain after transfer of inflammatory mediators/cytokines through the blood-brain barrier (67). Further, degradation of beneficial bacteria in the gut microbiota might result in a loss of microbiota-derived products such as butyrate which directly results in the downregulation of γ-aminobutyrate, serotonin and dopamine, all factors directly involved in the neurological regulation circuits and thus in the genesis of neuropsychiatric disorders when dysregulated (68).

Finally, to add another example to this collection, there is increasing evidence that similar mechanisms as involved in the protection from allergies might also play a role in the prevention of oncologic diseases (69). There is no doubt that preceding infections with certain pathogens may favor initiation and further development of several tumor disease entities. However, a variety of recent studies also demonstrated positive effects of pathogen-induced “benign” inflammatory processes on cancer development, even though the underlying mechanisms of this dichotomous influence of microbial exposure-mediated immune modulation on carcinogenesis are not well understood so far (70). As one example, the origins of childhood leukemia have long been discussed in the context of microbial stimuli in early childhood. Already at the end of 20^th^ century the question emerged whether early infections in childhood may act protectively against childhood acute leukemia by eliminating expanding aberrant leukocyte clones through well-trained and established immune mechanisms. In concordance with the Hygiene Hypothesis, Greaves propagated the “Delayed Infection Hypothesis” as an explanation for the development of childhood acute (lymphoblastic/myeloid) leukemia (ALL/AML) that peaks at the age of 2-5 years of life in affluent countries (71, 72). In his two hit model, Greaves proposed that based on a prenatally occurred chromosomal translocation or hyperdiploidy a pre-leukemic clone is already established around birth (first hit). A second hit event beyond the toddler age then leads to gene deletion or mutation and subsequent transformation to ALL/AML. While children suffering from infections and/or exposed to a rich microbial environment early in life might be ready to prevent that second aberration, predisposed children with an insufficiently educated immune system due to missing “old friends” contacts in the early postnatal life might not be able to eliminate expanding malignant cell clones (72). A number of studies aimed to prove this hypothesis by exploiting “day care attendance” before the third year of life as a proxy for infection. This concept is still a matter of debate. While the vast majority of these studies could add evidence to the Greaves hypothesis, some well-conducted studies could not support his assumptions (73, 74). Recently, a meta-analysis investigated the farm effect with regard to childhood leukemia and confirmed that contact to livestock provides protection not only against allergies but also against childhood leukemia (75). This study might point out to microbiota as a crucial player in both prevention of allergies and childhood cancer.

The challenges outlined in this mini review are intended to stimulate further exciting debates that might result in continuing revisions and adaptations of the Hygiene Hypothesis. We are aware that the examples reported in this review may only describe a limited subjective selection of the scientific topics currently discussed in context of the Hygiene Hypothesis. However, it is common to all topics that the explanations to unravel the underlying mechanisms refer to the close and beneficial relationship between man and microbes as established on the mucosal surfaces of our body. These interactions result in adequate shaping of adaptive systems of the body (mainly the immune system) that enables the whole organisms to appropriately handle diverse adverse influences. Without exaggeration, this finding might be considered one of the most fundamental insights of the life sciences within the last thirty years.

## Author Contributions

All authors contributed by writing and editing of the manuscript. All authors contributed to the article and approved the submitted version.

## Funding

Funded by the German Center for Lung Research (DZL). Open Access was funded by the Library of the Philipps University Marburg, Germany.

## Conflict of Interest

The authors declare that the research was conducted in the absence of any commercial or financial relationships that could be construed as a potential conflict of interest.
